# Mitochondrial GCN5L1 acts as a novel regulator for iron homeostasis to promote sorafenib sensitivity in hepatocellular carcinoma

**DOI:** 10.1186/s12967-024-05404-3

**Published:** 2024-06-25

**Authors:** Xiuya Hu, Peiyu Zhang, Sai Li, Jiaqi Zhang, Danni Wang, Zihan Wang, Lu Zhu, Lingdi Wang

**Affiliations:** 1 State Key Laboratory of Experimental Hematology, Tianjin Key Laboratory of Inflammatory Biology, Department of Physiology and Pathophysiology, School of Basic Medical Sciences, Tianjin Medical University, Main Bldg., R1306 22 Qixiangtai Rd, Tianjin, 300070 China; 2 The Province and Ministry co-sponsored Collaborative Innovation Center for Medical Epigenetics, NHC Key Laboratory of Hormones and Development, Chu Hsien-I Memorial Hospital and Tianjin Institute of Endocrinology, Department of Pharmacology, School of Basic Medical Sciences, Tianjin Medical University, Tianjin, China; 3 Department of Gynecology and Obstetrics, Tianjin Medical University General Hospital, Tianjin, China

**Keywords:** Sorafenib, Mitochondrial ROS, Mitochondrial iron, CISD1, GCN5L1, Hepatocellular carcinoma

## Abstract

**Background:**

Sorafenib resistance is becoming increasingly common and disadvantageous for hepatocellular carcinoma (HCC) treatment. Ferroptosis is an iron dependent programmed cell death underlying the mechanism of sorafenib. Iron is crucial for synthesis of cofactors essential to mitochondrial enzymes and necessary for HCC proliferation, while mitochondrial iron overload and oxidative stress are associated with sorafenib induced ferroptosis. However, the crosstalk among iron homeostasis and sorafenib resistance is unclear.

**Methods:**

We conducted bioinformatics analysis of sorafenib treated HCC datasets to analyze GCN5L1 and iron related gene expression with sorafenib resistance. GCN5L1 deleted HCC cell lines were generated by CRISPR technology. Sorafenib resistant HCC cell line was established to validate dataset analysis and evaluate the effect of potential target.

**Results:**

We identified GCN5L1, a regulator of mitochondrial acetylation, as a modulator in sorafenib-induced ferroptosis via affecting mitochondrial iron homeostasis. GCN5L1 deficiency significantly increased sorafenib sensitivity in HCC cells by down-regulating mitochondrial iron transporters CISD1 expression to induce iron accumulation. Mitochondrial iron accumulation leads to an acceleration in cellular and lipid ROS. Sorafenib resistance is related to CISD1 overexpression to release mitochondrial iron and maintaining mitochondrial homeostasis. We combined CISD1 inhibitor NL-1 with sorafenib, which significantly enhanced sorafenib-induced ferroptosis by promoting mitochondrial iron accumulation and lipid peroxidation. The combination of NL-1 with sorafenib enhanced sorafenib efficacy in vitro and in vivo.

**Conclusions:**

Our findings demonstrate that GCN5L1/CISD1 axis is crucial for sorafenib resistance and would be a potential therapeutic strategy for sorafenib resistant HCC.

**Supplementary Information:**

The online version contains supplementary material available at 10.1186/s12967-024-05404-3.

## Background

Hepatocellular carcinoma (HCC) is the second leading cause of malignant cancer-related mortalities, representing approximately 90% of all primary hepatic carcinomas worldwide [1]. The incidence and mortality of hepatocellular carcinoma (HCC) are rapidly increasing over time since patients are diagnosed in advanced stage while effective interventions are limited [2]. The liver is a critical hub for numerous physiological processes, including macronutrient metabolism, immune system support, glucose, and lipid metabolism [3]. Mitochondria are powerhouse of the cell, also serve as the defensive machinery maintaining cellular redox homeostasis in hepatocytes [4, 5]. Iron is the most prevalent metal in mitochondria, where iron is utilized for synthesis of cofactors essential to mitochondrial metabolic enzymes and supporting the electron transport chain to generate ATP, thus mitochondrial iron levels are tightly associated with mitochondrial energy metabolism [5]. Moreover, Iron plays an important role in cancer biology [6]. The negative effects of iron overload have been linked to HCC development [7]. Dysregulated iron metabolism has been identified as a metabolic hallmark of malignant cancer cells [8]. Iron deficiency inhibits HCC proliferation but promotes HCC progression and metastasis [9, 10], suggesting a critical role of iron dynamics in HCC progression.

Ferroptosis, a form of programmed cell death driven by iron-dependent lipid peroxidation which has emerged as a novel form of cell death, is highly relevant to chemotherapy of HCC [11, 12]. Oxidative stress and impaired redox homeostasis are necessary to induce ferroptosis [13]. Sorafenib, a ferroptosis inducer, is the first-line therapy for patients with HCC in advanced stage [14]. Unfortunately, most patients did not experience a long-term benefit because of early emergence of sorafenib resistance (Only approximately 30% of patients can benefit from sorafenib, and this population usually acquires drug resistance within half a year) [15]. The mechanisms underlying sorafenib resistance remain elusive. Emerging evidence show that iron deficiency mediating upregulation of HIF1-alpha leads to sorafenib resistance, suggesting a pivotal role of iron dynamics involving sorafenib sensitivity [16]. Iron homeostasis requires several transporters for iron entry and release from the cell and mitochondria, including TF/TFRC for iron uptake into the cell, MFRN1/MFRN2 for iron transporting into mitochondria, SLC40A1 and CISD1 for iron release from the cell and mitochondria [17]. CISD1 is a redox-sensitive protein with 2Fe-2S clusters to regulate mitochondrial iron content [18, 19], and plays a vital role in regulating lipid metabolism, cancer cell proliferation [20, 21], and erastin-induced ferroptosis [22]. However, as key organelles for cellular ROS generation and iron storage, the regulatory role of mitochondria in sorafenib resistance is elusive.

Protein acetylation is the major post translational modification in mitochondria, which modulates activities of mitochondrial metabolic enzymes in response to nutrient alteration as well as mitochondrial superoxide dismutase (SOD2) for antioxidant defense [23]. GCN5L1, is a mitochondrial enriched protein, and a small portion localizes in cytosol as well. GCN5L1 specifically modulates protein acetylation in mitochondria and modulates hepatic ROS generation to regulate gluconeogenesis [24]. Our previous work has identified that GCN5L1 loss promotes fatty acid oxidative to enhance ROS levels in HCC cells to facilitate cancer metastasis [25]. We aim to investigate mitochondrial acetylation associated metabolic reprogramming in the regulation of sorafenib sensitivity.

In the current study, we found GCN5L1 expression was associated with sorafenib sensitivity. Acute sorafenib treatment decreased GCN5L1 expression, while GCN5L1 is highly expressed in sorafenib resistant cells. GCN5L1 loss significantly increased sorafenib sensitivity of HCC cells, via induction of iron accumulation. GCN5L1 ablation inhibited transcriptional levels of CISD1 and SLC40A1 to block iron release, which resulted in lipid peroxidation to facilitate sorafenib-induced ferroptosis. Sorafenib resistance was associated with CISD1 overexpression. We analyzed Kaplan-Meier of liver cancer with sorafenib treatment and found that HCC patients with low GCN5L1 or CISD1 expression survived longer than those with high expression. The combination of CISD1 inhibitor NL-1 and sorafenib significantly enhanced the effect of sorafenib suggesting a potential therapeutical strategy for HCC.

## Methods

### Animals

Animal protocols were approved by the Animal Care and Use Committee of Tianjin Medical University. Animals were maintained in SPF environment with a 12-hour light/dark cycle and housed with free access to water and normal chow diet. C57BL/6J mice were purchased from Beijing Vital River Laboratory Animal Technology Co., Ltd. DEN-induced HCC model was generated as previously described [26]. Briefly, 2-week-old C57BL/6J wild-type mice were intraperitoneally administrated with DEN (25 mg/kg i.p.), followed by CCl_4_ (5 ml/kg i.p., 10% dissolved in olive oil) injection weekly for 10 times, started at 4 weeks of age [27]. For in-vivo treatment of sorafenib and NL-1, the mice were randomly separated into four groups (10mL/kg saline, 30 mg/kg sorafenib, 10 mg/kg NL-1 and sorafenib plus NL-1). The mice were intravenously injected with relative chemicals 6 times a week for two weeks and then sacrificed.

### Cell culture and transfection

HepG2, Huh7, Hep3B and HEK293T cells were obtained from the American Type Culture Collection (ATCC). The cells were cultured in DMEM (Meilun Biotechnology, Cat# MA0212) supplemented with 10% fetal bovine serum plus 1% antibiotics at 37 °C with 5% CO_2_. For cell transfection, packaging plasmids and expression plasmids (sg-GCN5L1, sg-Ctrl, Vector, MtG-Myc and CISD1-Flag) were transfected into HEK293T cells. The virus was collected after 48 h and passed through 0.45 μm filter. After infected with the virus, HCC cells were maintained in medium containing Puromycin (2 μg/mL, Solarbio) to generate stable GCN5L1 KO, MtG overexpression and CISD1 overexpression cells.

For establishment of sorafenib resistant HepG2 cells, we treated HepG2 with increasing sorafenib concentration at the starting concentration of 0.5 μM. Cells were maintained in each concentration of sorafenib for 2 weeks, followed by increasing the concentration of sorafenib by 1 μM, until that the cells could survive from higher concentration of sorafenib treatment. Resistant cells were confirmed by IC50 measurement.

### Western blotting assay

The cells were treated with indicated drugs after being seeded for 24 h. The total protein was extracted by RIPA lysate and the concentration of protein was determined by using BCA (Beyotime, Jiangsu, China). Equal amounts of protein (50 μg) were then electrophoresed by SDS–PAGE gels (US EVERBRIGHT, Suzhou, China), which were then transferred to PVDF membranes (Millipore Corp, Billerica, MA, USA). The membranes were blocked with skim milk and incubated with the primary antibody. Next, after incubating with the secondary antibody, we use an enhanced chemiluminescence (ECL) Kit (Thermo Scientific, USA) to detect the expression of proteins and recorded them with the chemiluminescence imaging system (MINICHEMI). The following antibodies were used: anti-β-Actin (1:1000, Cell Signaling Technology, Cat# 4967 S), anti-GCN5L1 (1:1000, ABclonal, customization), anti-CISD1 (1:10000, Proteintech, Cat# 16006-1-AP), anti-SLC40A1 (1:1000, Proteintech, Cat# 26601-1-AP).

### Cell viability assay

For assessment of cell viability, HCC cells were seeded in 96-well plates at a density of 3000 cells per well and treated with doses of sorafenib (Selleck, Cat# S1040), Fer-1 (5 μM, Selleck, Cat# S7397), NL-1 (20 μM, Selleck, Cat# S0767), DFO (50 μM, Selleck, Cat# S5742), Z-VAD-FMK (50 μM, MedChemExpress, Cat# 161401-82-7) Necrostatin-1 (50 μM, MedChemExpress, Cat# 4311-88-0) or pioglitazone (10 μM, Selleck, Cat# S2046) for 48 h at 37℃. Next, the cells were treated with 100 μL MTT reagent (5 mg/mL, Solarbio, Cat# M8180) for 4 h and measured the absorbance at 490 nm by using a microplate reader.

### Colony formation assay

For colony formation assay, HCC cells were seeded in 96-well plates at a density of 3000 cells per well and treated with different concentrations of sorafenib or NL-1 for 2 weeks. Then, the cells were washed with phosphate-buffered saline (PBS), fixed with 4% paraformaldehyde and stained with 0.1% crystal violet solution (Solarbio, Cat# G1064) for 30 min, imaged and counted.

### Quantitative real-time polymerase chain reaction

Total RNA of cells was extracted using Trizol RNA isolation reagent (Invitrogen, California, USA), and TransScript^II^ First Strand cDNA Synthesis SuperMix (Yeasen, Shanghai, China) was used to synthesize cDNA according to the manufacturer’s instructions, quantitative RT-PCR analysis was then performed using SYBR Green PCR Mix (Yeasen, Shanghai, China) and target gene-specific primers. Data were normalized to the mRNA levels of β-Actin in each individual sample. RT-PCR primers are shown in Supplementary Table 1.

### ROS analysis

Reactive oxygen species (ROS) levels were measured by using the DCFH-DA (Beyotime, Jiangsu, China). HepG2 cells in 96-well plates were stained with the fluorescent probe DCFH-DA (10 μM) for 20 min at 37 ℃, next the cells were washed with PBS for three times, and the fluorescence intensity was measured by microplate reader according to the instruction [28]. The final results were obtained by normalizing the fluorescence intensity to protein contents.

Flow cytometry was applied to analyze the production of ROS in HepG2 cells. Cells were seeded in 6-well plates at a density of 1 × 10^5^ cells per well and treated with or without sorafenib (8 μM, 12 h). The cells were incubated with DCFH-DA (10 μM) at 37 °C for 20 min, followed by collecting cells in PBS. 10,000 cells per sample were analyzed on a BD FACSVerse flow cytometer using an argon-ion laser (15 mWatt) with incident beam at 488 nm.

### Intracellular iron assay

For intracellular iron assay, HepG2 cells were seeded in confocal chambers and treated with series concentrations of drugs, then FerroOrange (1 μM, DOJINDO, Kyushu, Japan) and Mito-FerroGreen (5 μM, DOJINDO, Kyushu, Japan) were applied to detect the intracellular and mitochondrial iron levels according to the manufacturers’ instructions. The fluorescence signals were imaged by fluorescence microscope (Carl Zeiss AG, Oberkochen, Germany) and fluorescence intensity was measured by ImageJ software. The mean fluorescence intensity of each group was normalized to the control group. Colorimetric methods were used to detect iron contents in cell lysates or isolated mitochondria by the commercial kit (Beyotime, Jiangsu, China).

### Detection of lipid peroxidation

C11-BODIPY^581/591^ (5 μM, Thermo Fisher Scientific, Massachusetts, USA, Cat# D3861) was used to detect lipid ROS. Cells seeded in glass bottom cell culture dishes with indicated treatment were incubated with C11-BODIPY^581/591^ (5 μM) for 30 min at 37 °C and washed three times with PBS. Finally, the fluorescence was detected by confocal microscope (Carl Zeiss AG, Oberkochen, Germany) and fluorescence intensity of each cell was measured by ImageJ software. At least 50 cells per group were calculated the FITC/PE ratio to indicate lipid oxidation and the mean ratio of each group was normalized to the control group.

### Mitochondria isolation

Cells were homogenized in mitochondrial isolation buffer (225mM mannitol, 75mM sucrose, 0.5% BSA, 0.5 mM EGTA, and 30mM Tris-HCl PH 7.4), mitochondria were then collected by two-step centrifugation at 600 g to remove intact cells and debris. Finally, 9000 g to concentrate mitochondria and separate them from other organelles.

### Survival analysis

The effects of GCN5L1 or CISD1 on the survival of patients with liver cancer with sorafenib treatment were performed using the Kaplan Meier Plotter online survival analysis tool (https://kmplot.com/analysis/).

### Statistical analysis

Statistical analysis was performed with GraphPad Prism 8.0 software (San Diego, CA, USA). All data are reported as mean ± SEM from at least three independent experiments. The two-tailed unpaired student’s t-test or ANOVA plus a Bonferroni post hoc test were used to assess the differences between two or more groups. *p* < 0.05 indicates statistical significance.

## Results

### GCN5L1 deletion increases sensitivity of HCC to sorafenib via ferroptosis

**Fig. 1:**
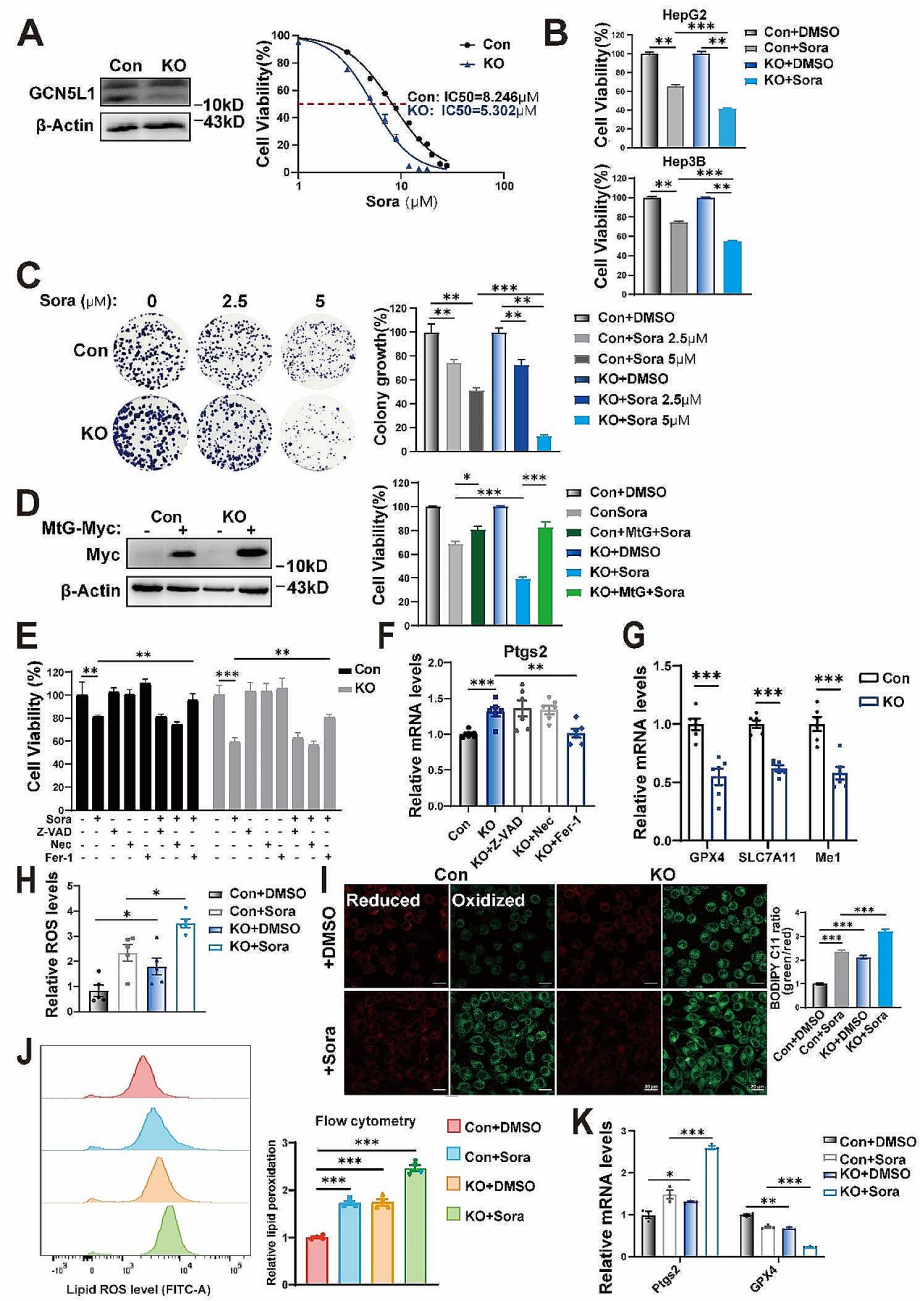
GCN5L1 deletion increases the sensitivity of HCC to sorafenib via ferroptosis. (**A**) GCN5L1 protein level in HepG2 Con and KO cells was confirmed by western blot (left). The IC50 values of HepG2 cells treated with sorafenib (right). (**B**) The MTT assay to analyze cell viability following sorafenib (8 μM) treatment for 48 h in HepG2 or Hep3B cells with GNC5L1 KO and control. (**C**) The colony formation assay showed the growth effect of GCN5L1 in HepG2 cells compared with the parental cells for 14 days as well as quantification of clusters (right). The values of each Con and KO group are normalized to their individual vehicle (DMSO) group. (**D**) Overexpression of mitochondrial located GCN5L1 was confirmed by immunoblot assay. (**E**) Cell viability of sorafenib (8 μM) alone or combined with necroptosis inhibitor necrostatin-1(Nec, 50 μM); pyroptosis and apoptosis inhibitor Z‐VAD (50 μM); ferrostatin‐1 (10 μM) on HepG2 cells. (**F**) RT-PCR analyses of ferroptosis relative gene Ptgs2 in HepG2 cells treated with Nec, Z-VAD, Fer-1. (**G**) The mRNA expression of ferroptosis-related genes was shown in indicated HepG2 cells (*n* = 6). (**H**) The ROS levels in indicated HepG2 cells were assayed by DCFH-DA staining (*n* = 5). (**I**) Live-cell fluorescence imaging of lipid peroxidation stained by C11-BODIPY in HepG2 cells and relative fluorescence intensity was quantified by ImageJ software. Oxidized C11-BODIPY is in green, a reduced C11-BODIPY is in red. Scale bar, 20 μm. (**J**) C11-BODIPY was measured by flow cytometry. (**K**) RT-PCR analyses of ferroptosis relative genes in HepG2 cells treated with or without sorafenib (8 μM) for 24 h. DMSO dimethyl sulfoxide, Sora sorafenib, Fer-1 ferrostatin-1, ROS reactive oxygen species, MtG mitochondrial localized GCN5L1. Values represent the mean ± SEM of three or more independent experiments. The two-tailed unpaired student’s t-test or ANOVA plus a Bonferroni post hoc test were used to assess the differences between two or more groups, *p* < 0.05 indicates statistical significance. *p < 0.05, **p < 0.01 ***p < 0.001

Ferroptosis is the underlying mechanism contributed to sorafenib-induced HCC cell death, which requires ROS generation, inhibition of redox system and iron overload [29]. GCN5L1 is a mitochondrial enriched protein, regulates mitochondrial protein acetylation and liver metabolism [24]. Our previous work has shown that GCN5L1 deletion enhances fatty acid oxidation in HCC cells leading to an increase of mitochondrial ROS, thereby promoting HCC metastasis [25]. Thus, GCN5L1 deficiency might influence sorafenib-induced ferroptosis. To explore this, we firstly evaluated the correlation of GCN5L1 expression with sorafenib sensitivity by analyzing sorafenib-resistant datasets of GSE62813 and GSE128683 and found a significant increase in GCN5L1 transcription levels in sorafenib-resistant HepG2 cells (Supplementary Fig. 1A). To confirm this, we established sorafenib resistant HepG2 cells (Supplementary Fig. 1B), which exhibited IC50 value of 16.51 μM comparing to sensitive cells with IC50 value of 7.56 μM (Supplementary Fig. 1C). We found that GCN5L1 protein levels were significantly increased in sorafenib resistant HepG2 cells compared to sensitive cells, consistent with dataset analysis (Supplementary Fig. 1D). Moreover, we analyzed the survival of HCC patients with sorafenib treatment and found that patients with low GCN5L1 expression survived longer than those with high GCN5L1 expression (Supplementary Fig. 1E). These data suggested that GCN5L1 expression was associated with sorafenib resistance. Interestingly, GCN5L1 protein levels were observed to be inversely correlated with sorafenib acute treatment, which represented a sensitive status of HepG2 to sorafenib (Supplementary Fig. 1F-G). Based on these results, we hypothesized that GCN5L1 deletion might increase the sensitivity of HCC to sorafenib. To test this hypothesis, we examined sorafenib sensitivity in GCN5L1 deletion (KO) and control (Con) HepG2 cells. GCN5L1 KO cells exhibited lower IC50 to sorafenib compared with the Con cells, suggesting that GCN5L1 deletion enhanced the sensitive to sorafenib (Fig. 1A). In parallel, we found that GCN5L1 deletion enhanced the effect of sorafenib in HepG2 and Hep3B cells (Fig. 1B). To confirm the function of GCN5L1 deletion on sorafenib sensitivity, we used colony formation assay with low dose of sorafenib to evaluate the sensitivity. We found that low dose of sorafenib (2.5 μM or 5 μM) slightly reduced colony formation in Con cells, the effects were dramatically enhanced in KO cells (Fig. 1C). GCN5L1 is a ubiquitous expression protein and enriched in mitochondria. In our previous work, we found mitochondrial localized GCN5L1 (MtG, GCN5L1 fused with mitochondrial targeting sequence) regulates ROS generation via modulating fatty acid oxidation (FAO) [25]. Therefore, we introduced MtG into HepG2 cells to investigate its function. MtG overexpression slightly inhibited sorafenib-induced cell death in HepG2 cells, meanwhile, restoration of MtG in KO cells reversed GCN5L1 deletion induced sorafenib sensitivity, implicating mitochondrial localized GCN5L1 plays a crucial role in sorafenib sensitivity, which could be related to ROS generation (Fig. 1D). Given that, sorafenib induced cell death includes apoptosis, ferroptosis, and necroptosis, to confirm the type of cell death pathway regulated by GCN5L1 deletion, we incubated HepG2 cells with sorafenib and cell death inhibitors. Ferrostatin-1 (Fer-1, ferroptosis inhibitor), but not Z-VAD-FMK (Z-VAD, apoptosis and pyroptosis inhibitor) or necrostatin-1 (Nec, necroptosis inhibitor) reversed sorafenib induced cell death in both GCN5L1 deletion and control cells (Fig. 1E). Moreover, PTGS2, which is considered as a marker of ferroptosis, was increased in GCN5L1 KO cells and restored by Fer-1 treatment (Fig. 1F). The data suggests ferroptosis was the major cell death pathway mediating sorafenib induced cell death in GCN5L1 ablation.

Cellular redox homeostasis is dynamic and maintained by the balance between the generation of oxidants and local antioxidative defense [30]. Impaired antioxidant system is not able to eliminate excessive ROS, which leads to ferroptosis [31]. We examined antioxidant related gene expressions and found the transcriptional levels of GPX4, SLC7A11, and ME1 were significantly decreased in GCN5L1 KO cells (Fig. 1G). In consideration of the importance of ROS in ferroptosis, we measured cellular ROS levels and found sorafenib induced cellular ROS, which was exacerbated by GCN5L1 deletion (Fig. 1H). We then determined lipid peroxidation by C11-BODIPY staining, which is used as a ferroptosis marker, and observed a consistent result as cellular ROS (Fig. 1I-J). GPX4 and PTGS2, markers of ferroptosis, were changed by GCN5L1 deletion and exacerbated in sorafenib treated KO cells (Fig. 1K). Interestingly, the basal levels of cellular ROS and lipid peroxidation were significantly increased in GCN5L1 KO cells, however, GCN5L1 deletion has been shown to promote HCC proliferation and metastasis [25, 26], therefore, GCN5L1 deletion induced lipid peroxidation was not sufficient to induce cell death but give a standby status for sorafenib-induced ferroptosis. Taken together, high expression of GCN5L1 is associated with sorafenib resistance in HCC, while GCN5L1 deletion increases sensitivity of HCC to sorafenib via ferroptosis.

### Mitochondrial GCN5L1 regulates iron homeostasis

**Fig. 2:**
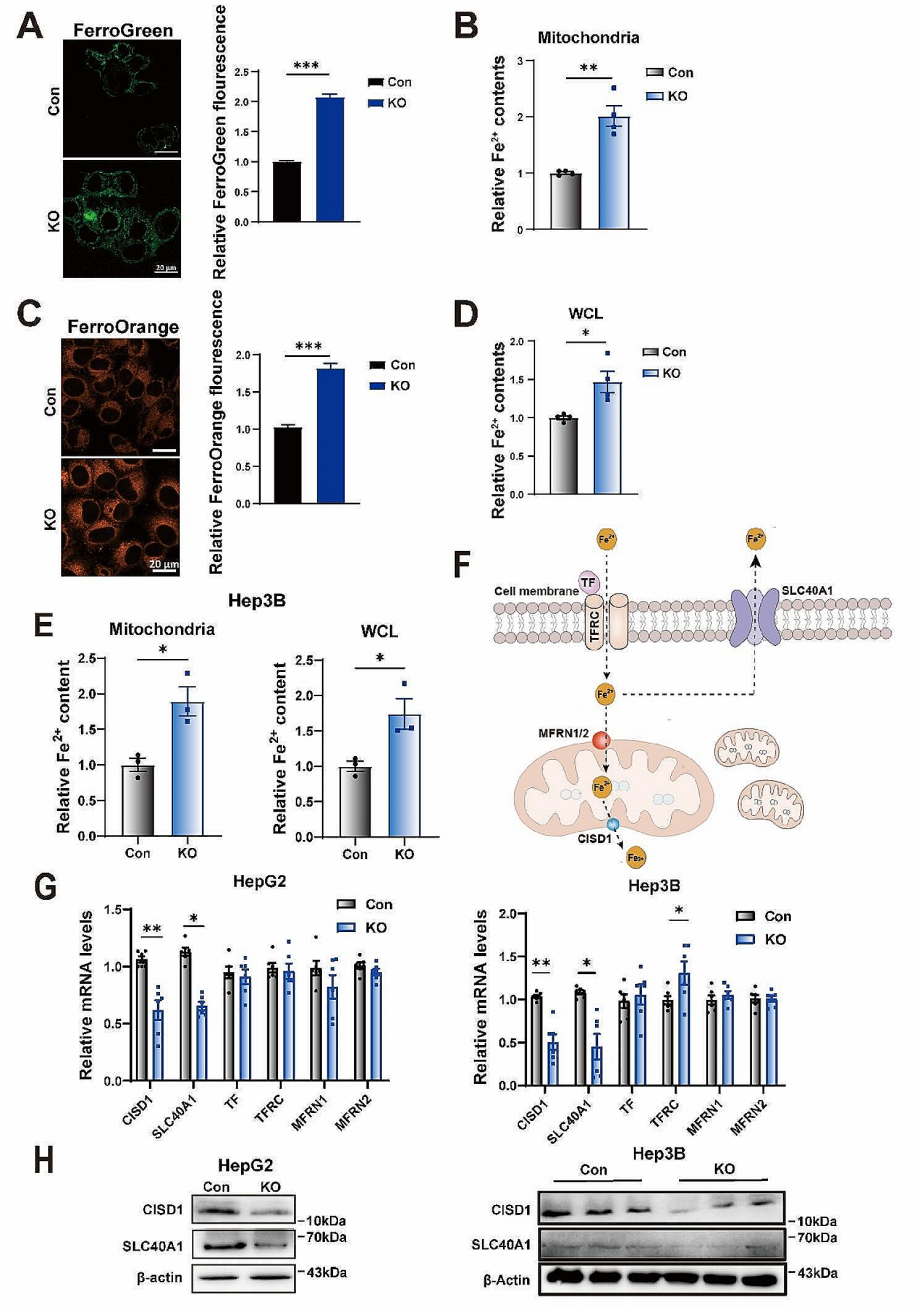
Mitochondrial GCN5L1 regulates iron homeostasis. (**A**) Mitochondrial Fe^2+^ was assayed by Mito-FerroGreen staining (left) and relative fluorescence intensity of Mito-FerroGreen (right) was quantified in HepG2. The fluorescence intensity was quantified by ImageJ software. Scale bar, 20 μm. (**B**) The mitochondrial iron levels in Con and KO cells of HepG2 (*n* = 4). (**C**) Intracellular Fe^2+^ was detected by FerroOrange staining (left) in HepG2 cells and relative fluorescence intensity of FerroOrange was quantified (right). Scale bar, 20 μm. (**D**) The intracellular total iron levels in Con and KO cells (*n* = 4). (**E**) The mitochondrial and intracellular iron levels in Hep3B cells with GCN5L1 KO and control (*n* = 3). (**F**) Iron transporter gene in HepG2 cells. (**G**) RT-qPCR analyses of iron transporter gene expression in HepG2/Hep3B Con and KO cells (*n* = 6). (**H**) Western Blotting analysis of iron transporter proteins of HepG2/Hep3B Con and KO cells. DMSO dimethyl sulfoxide, Sora sorafenib, Fer-1 ferrostatin-1, ROS reactive oxygen species. Values represent the mean ± SEM of three or more independent experiments. Statistical significance was calculated using two-tailed unpaired Student’s t-test. *p < 0.05, **p < 0.01 ***p < 0.001

To investigate the mechanism that mitochondrial restricted GCN5L1 regulates lipid peroxidation and sorafenib sensitivity, we performed immunoprecipitation coupled mass spectrometry (IP-MS) to identify potential proteins interaction with MtG. IP-MS data revealed that the mitochondrial outer membrane protein CISD1 was a potential target of GCN5L1 (Supplementary Tables 2 and Supplementary Fig. 2A). CISD1 functions as an iron transporter which governs iron excretion from mitochondria to cytosol [32]. Iron deficiency is associated with sorafenib resistance in HCC [16], moreover, CISD1 has been reported to be induced by erastin to inhibit ferroptosis by protection against mitochondrial lipid peroxidation [22]. We hypothesized that GCN5L1 interacts with and regulates CISD1 activity to modulate iron homeostasis and sorafenib-induced ferroptosis. To test this, we utilized the fluorescent probes Mito-FerroGreen and FerroOrange to evaluate mitochondrial and cellular iron contents in HepG2 cells. Mitochondrial iron contents were significantly increased in GCN5L1 KO cells compared with control cells (Fig. 2A). This result was confirmed by colorimetric method measurement (Fig. 2B). Intracellular Iron accumulation was similar to that of mitochondrial iron contents in GCN5L1 KO cells (Fig. 2C-D). In parallel, mitochondrial and cellular iron contents were increased in GCN5L1 deleted Hep3B cells (Fig. 2E). The intracellular labile iron pool (LIP) is maintained by several transporters regulating iron enter and release from the cell and mitochondria, including transferrin (TF) / transferrin receptor 1(TFR1) for iron uptake into cytosol, SLC40A1 for iron release, Mitoferrin (MFRN1/2) and CISD1 for iron transfer from mitochondria (Fig. 2F) [17]. To investigate the mechanism of GCN5L1 loss induced iron accumulation, we first examined the transcription levels of these typical iron transporters and found that CISD1 and SLC40A1 were significantly reduced in GCN5L1 KO cells (Fig. 2G), which was coincident with iron accumulation in GCN5L1 KO cells. We then confirmed the reduced protein levels of iron release transporters in GCN5L1 deleted HCC cells (Fig. 2H). These data suggest that GCN5L1 deletion suppressed the expression of iron release transporters to enhance iron accumulation, which could promote lipid peroxidation and make HCC cells be standby for sorafenib-induced ferroptosis.

We then analyzed the expression of iron transporters in sorafenib resistant HepG2 cells. Dataset analysis revealed that sorafenib resistance significantly altered the expression of iron transport related genes (Supplementary Fig. 2B), which might be associated with iron deficiency in sorafenib resistance [16]. Moreover, increased CISD1 protein levels were observed in sorafenib resistant HepG2 cells (Supplementary Fig. 2C). In parallel, HCC patients with high expression of CISD1 revealed worse survival with sorafenib treatment compared to the ones with low levels of CISD1, suggesting CISD1 expression and mitochondrial iron depletion were associated with sorafenib resistance (Supplementary Fig. 2D). We then measured mitochondrial and cellular iron contents in sorafenib sensitive and resistant HepG2 cells and observed decreased iron contents in sorafenib resistant cells (Supplementary Fig. 2E). Sorafenib resistant HepG2 cells exhibited high expression of GPX4 and SLC7A11, suggesting increased antioxidant capacity was coincident with iron homeostasis and sorafenib resistance (Supplementary Fig. 2F). Together, sorafenib resistance was associated iron deficiency might be related to the inhibition of iron uptake and activation of iron release, while GCN5L1 deletion suppressed the expression of iron release transporters to enhance iron accumulation thereby promoting sorafenib-induced ferroptosis.

### Iron accumulation promotes KO cells to sorafenib-induced ferroptosis

**Fig. 3:**
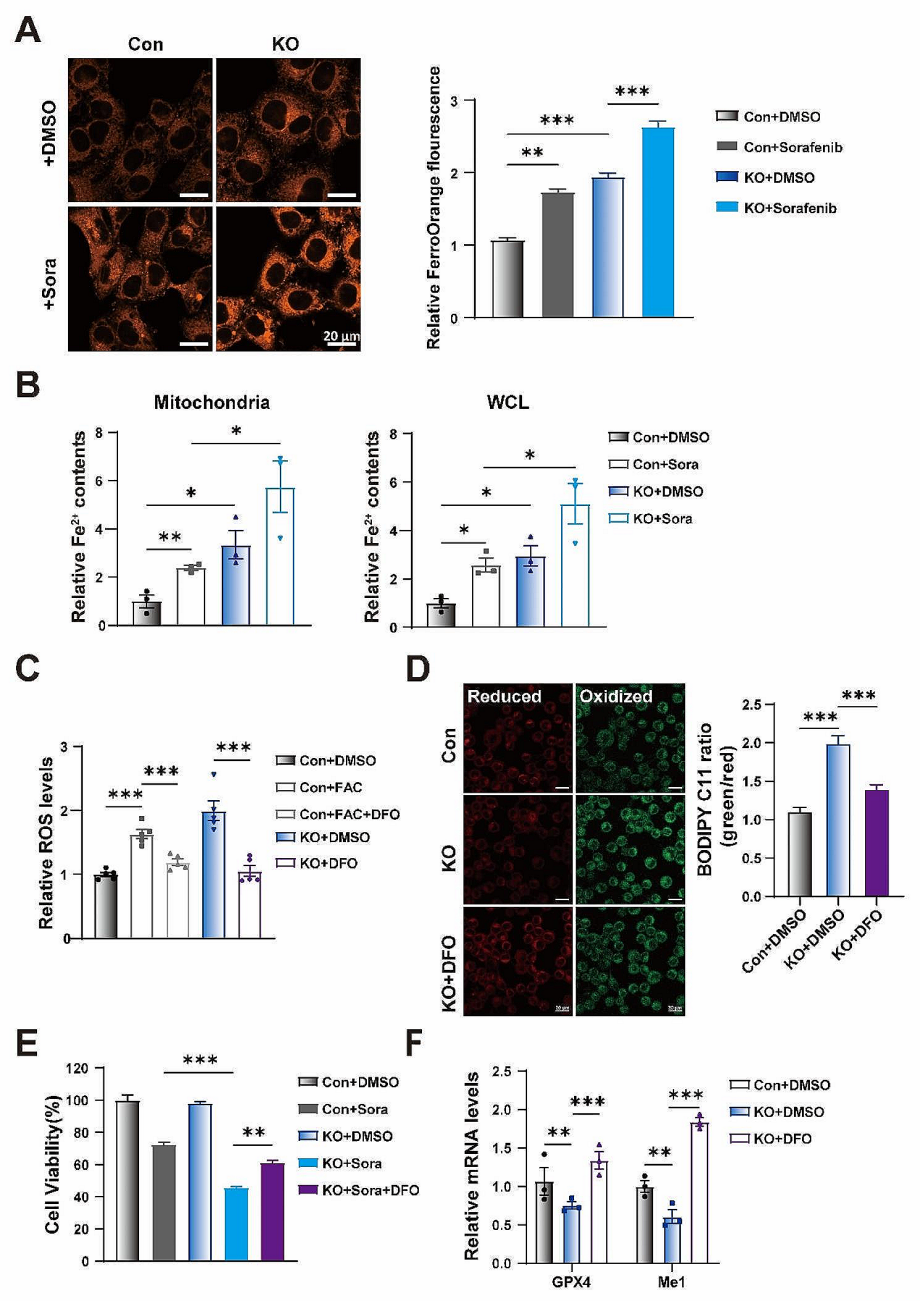
Iron metabolism disturbance promotes KO cells to sorafenib-induced ferroptosis. (**A**) Representative images of FerroOrange of HepG2 cells with or without sorafenib treatment are shown (left) and the fluorescence intensity was quantified by ImageJ software (right). Scale bar, 20 μm. (**B**) Cellular mitochondrial iron level (left) and total iron level (right) after 24 h of sorafenib (5 μM) treatment (*n* = 3). (**C**) The ROS levels in indicated HepG2 cells treated with or without FAC (100 μM) and DFO (50 μM) for 12 h were assayed by DCFH-DA staining (*n* = 5). (**D**) Representative images of the fluorescent probe C11-BODIPY and relative fluorescence intensity was quantified by ImageJ software. Scale bar, 20 μm. (**E**) Cell viability was assayed in HepG2 cells treated with or without sorafenib (8μM) and DFO (50 μM) for 48 h. (**F**) Expression of GPX4 and Me1 mRNA in HepG2 cells treated with or without DFO(50 μM) for 24 h was measured by RT-PCR (*n* = 3). DMSO dimethyl sulfoxide, Sora sorafenib, FAC ammonium iron citrate (Fe^3+^), DFO deferoxamine, ROS reactive oxygen species. Values represent the mean ± SEM of three or more independent experiments. The two-tailed unpaired student’s t-test or ANOVA plus a Bonferroni post hoc test were used to assess the differences between two or more groups, *p* < 0.05 indicates statistical significance. *p < 0.05, **p < 0.01 ***p < 0.001

Cancer cells have an increased demand for iron to support their growth. This dependence on iron makes cancer cells more susceptible to iron-induced cell death [33]. GCN5L1 KO cells exhibited increased iron content, which could make GCN5L1 KO cells susceptible to sorafenib. Therefore, targeting iron homeostasis could become a novel approach to re-sensitize drug-resistant cancer cells. The FerroOrange staining and colorimetric measurements showed that mitochondrial and cellular iron contents were significantly enhanced by sorafenib incubation, and GCN5L1 deletion exacerbated iron accumulation upon sorafenib incubation (Fig. 3A-B). This data suggested that GCN5L1 might modulate iron transporting, possessing a different regulatory pathway of sorafenib, to enhance sorafenib-induced ferroptosis. To evaluate the role of iron in the regulation of ferroptosis, we incubated HepG2 cells with FAC (Fe^3+^) and found cellular ROS was significantly enhanced, which was reversed by iron chelator DFO (deferoxamine) (Fig. 3C). Again, DFO reversed GCN5L1 KO induced cellular ROS (Fig. 3C), suggesting iron accumulation could be the pathway mediating FAO to induce ROS levels in GCN5L1 KO cells. Lipid peroxidation assay by C11-BODIPY staining showed a consistent result that DFO inhibited lipid peroxidation in GCN5L1 KO cells (Fig. 3D). DFO partially reversed sorafenib-induced cell death in GCN5L1 KO cells, implying iron accumulation contributed to GCN5L1 deletion induced sorafenib sensitivity (Fig. 3E). Again, we examined ferroptosis related gene expression, and observed that the expression of GPX4 and ME1 was reversed by DFO in GCN5L1 KO cells (Fig. 3F). Taken together, the data supported that the iron accumulation contributed to cellular oxidative stress, which increased the sensitivity of GCN5L1 deleted HCC cells to sorafenib.

### Restoration of CISD1 reverses sorafenib sensitivity in GCN5L1 KO cells

Acute sorafenib treatment induced iron accumulation (Fig. 3A-B), which is necessary for ferroptosis [33]. However, sorafenib resistance is shown to be associated with highly expressed CISD1 as along with iron deficiency (Supplementary Fig. 2) [16, 34]. Therefore, acute sorafenib treatments may regulate the expression or activities of CISD1 and SLC40A1 to influence iron accumulation and oxidative stress. HepG2 cells were incubated with sorafenib for 24 h to 48 h and at doses of 5 μM to 10 μM. Sorafenib inhibited CISD1 expression in time- and dose-dependent manners (Supplementary Fig. 3A-B). Increased cellular iron levels by FAC incubation inhibited CISD1 expression (Supplementary Fig. 3C). Iron chelator DFO reversed sorafenib-induced CISD1 loss (Supplementary Fig. 3D). The data suggested that the mitochondrial iron transporter CISD1 was susceptible to acute sorafenib treatment, which might be feedback regulated by cellular iron content or ROS. Furthermore, we found that both Fer-1 and DFO restored CISD1 expression in KO cells (Supplementary Fig. 3E), suggesting ROS and iron levels were involved in CISD1 transcriptional regulation. Reversed CISD1 expression in GCN5L1 KO cells by MtG overexpression confirmed the regulation of CISD1 expression by GCN5L1 deletion (Supplementary Fig. 3F). We then introduced Mito-TEMPO or N-acetylcysteine (NAC), a mitochondrial or cellular ROS scavenger, to treat GCN5L1 KO cells and found reduction of mitochondrial ROS reversed CISD1 expression in GCN5L1 KO cells, supporting that CISD1 expression is regulated by ROS (Supplementary Fig. 3G).

**Fig. 4:**
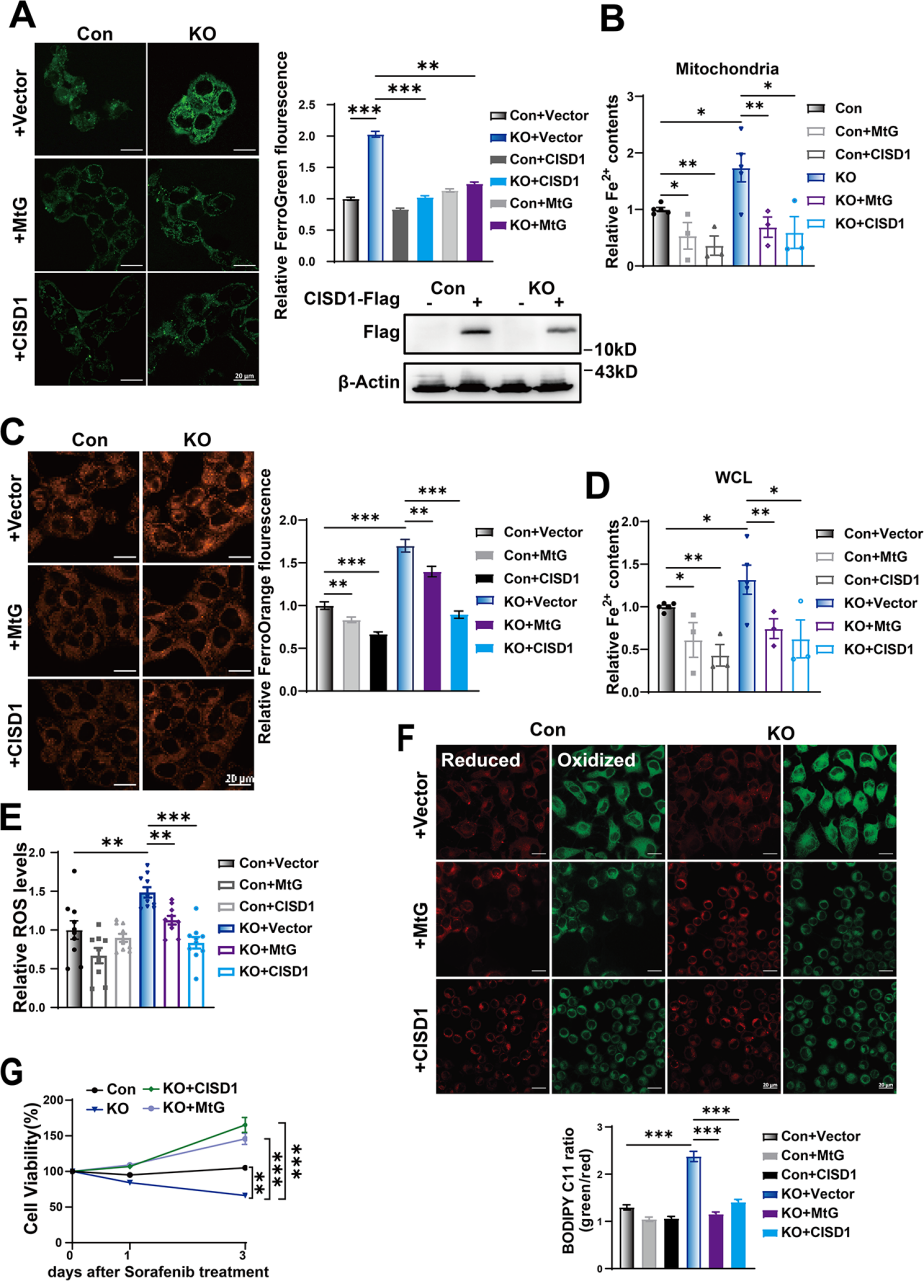
Restoration of CISD1 reverses sorafenib sensitivity in GCN5L1 KO cells. (**A**) Overexpression of CISD1 was confirmed by immunoblot assay (right). Mitochondrial Fe^2+^ assayed by Mito-FerroGreen (left) and relative fluorescence intensity (right) in HepG2 cells transfected as indicated was quantified by ImageJ software. Scale bar, 20 μm. (**B**) The mitochondrial iron levels in HepG2 cells transfected as indicated (*n* = 3–4). (**C**) Intracellular Fe^2+^ detected by FerroOrange and relative fluorescence intensity of FerroOrange in HepG2 cells transfected as indicated. Scale bar, 20 μm. (**D**) The intracellular total iron levels in transfected HepG2 cells (*n* = 3–4). (**E**) The ROS levels in transfected HepG2 cells were assayed by DCFH-DA staining (*n* = 10). (**F**) C11-BODIPY staining of transfected HepG2. Statistical analysis of cell fluorescence of Bodipy is shown (below). Scale bar, 20 μm. (**G**) The MTT assay to analyze the viability of transfected cells following sorafenib (5 μM) treatment (*n* = 3). MtG mitochondrial located GCN5L1, ROS reactive oxygen species. Values represent the mean ± SEM of three or more independent experiments. The two-tailed unpaired student’s t-test or ANOVA plus a Bonferroni post hoc test were used to assess the differences between two or more groups, *p* < 0.05 indicates statistical significance. *p < 0.05, **p < 0.01 ***p < 0.001

CISD1 has been shown to be critical for erastin-induced ferroptosis [22]. We speculated that GCN5L1 KO induced cellular and mitochondrial iron accumulation and sensitivity to sorafenib might be influenced by CISD1 expression. We introduced CISD1 overexpression in HepG2 cells and found CISD1 reduced mitochondrial iron contents in GCN5L1 KO cells, while MtG overexpression exhibited similar results (Fig. 4A-B). Cellular iron contents exhibited consistent results (Fig. 4C-D). Cellular ROS and lipid peroxidation levels showed that CISD1 overexpression significantly reduced ROS and lipid peroxidation in GCN5L1 KO cells, suggesting the important role of CISD1 in GCN5L1 KO cells mediating the regulation of ROS and lipid peroxidation (Fig. 4E-F). In addition, restoration of CISD1 completely inhibited GCN5L1 deletion induced sorafenib sensitivity, implying that reduced CISD1 expression was the underlying mechanism of GCN5L1 loss induced sorafenib sensitivity (Fig. 4G). Together, CISD1 expression was associated with cellular ROS and iron content, whose reduction led to increased sorafenib sensitivity in GCN5L1 KO cells.

### CISD1 inhibitor accelerates sorafenib-induced HCC ferroptosis

**Fig. 5:**
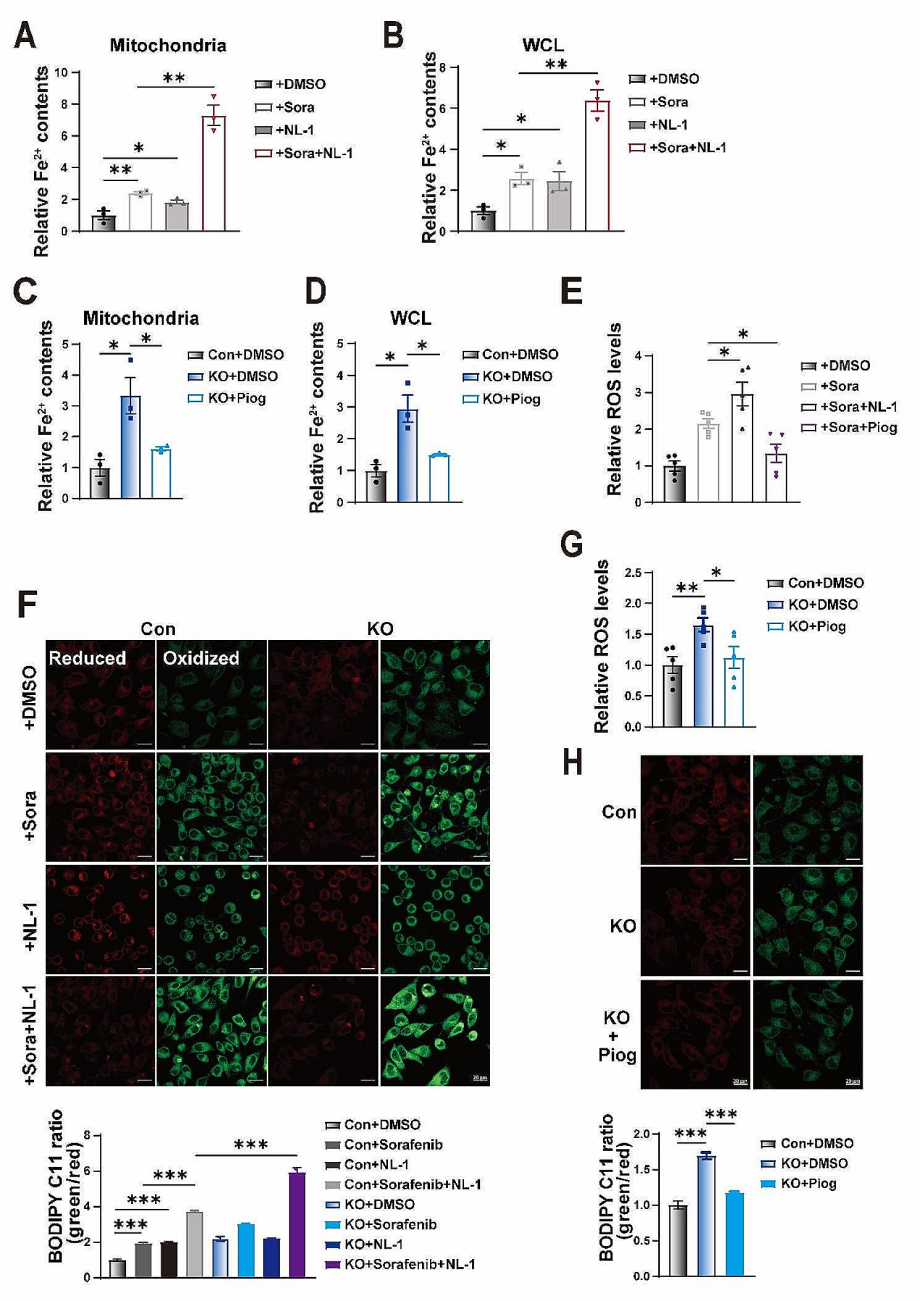
CISD1 inhibitor accelerates sorafenib-induced HCC ferroptosis. (**A**-**B**) The intracellular mitochondrial and total iron levels in HepG2 cells treated with or without sorafenib (5 μM) and NL-1 (20 μM) for 24 h (*n* = 3). (**C**-**D**) The intracellular mitochondrial and total iron levels in HepG2 cells treated with or without pioglitazone (10 μM) for 24 h (*n* = 3). (**E**) The ROS levels in indicated HepG2 cells treated with or without sorafenib (5 μM), NL-1 (20 μM) and pioglitazone (10 μM) for 12 h were assayed by DCFH-DA staining (*n* = 5). (**F**) Fluorescence images of C11-BODIPY-stained HepG2 cells cultured in medium supplemented with individual or combination of 5 μM sorafenib and 20 μM NL-1 for 24 h. The relative fluorescence intensity was quantified by ImageJ software. Green, C11-BODIPY (oxidized). Scale bar, 20 μm. (**G**) The ROS levels in indicated HepG2 cells treated with or without pioglitazone (10 μM) for 12 h were assayed by DCFH-DA staining (*n* = 5). (**H**) Fluorescence images of C11-BODIPY-stained HepG2 cells cultured in medium supplemented with or without 10 μM pioglitazone for 24 h and relative fluorescence intensity was quantified by ImageJ software. Scale bar, 20 μm. DMSO dimethyl sulfoxide, Sora sorafenib, Piog pioglitazone. Values represent the mean ± SEM of three or more independent experiments. The two-tailed unpaired student’s t-test or ANOVA plus a Bonferroni post hoc test were used to assess the differences between two or more groups, *p* < 0.05 indicates statistical significance. *p < 0.05, **p < 0.01 ***p < 0.001

Mitochondrial iron content is critical for sorafenib-induced ferroptosis, and CISD1 expression is highly responsible for sorafenib treatment. CISD1 attenuated the intracellular oxidative stress response, and we found that inhibition of CISD1 promoted sorafenib-induced ferroptosis. NL-1 has been reported to be an inhibitor of CISD1 which mediates autophagy and increases NL-1 induced tumor cell death [35]. We hypothesized that inhibition of CISD1 by NL-1 might enhance sorafenib sensitivity or attenuate sorafenib resistance. As control, we utilized pioglitazone, which is an activator of CISD1 for promoting mitochondrial iron release, inhibiting of lipid peroxidation and subsequent ferroptosis [22]. We first measured mitochondrial and cellular iron contents in HepG2 cells with sorafenib or NL-1 incubation for 24 h. We found that NL-1 significantly increased mitochondrial and cellular iron contents (Fig. 5A-B). Interestingly, NL-1 significantly promoted sorafenib-induced mitochondrial and cellular iron accumulation, suggesting that the combination of sorafenib and NL-1 might enhance sorafenib-induced ferroptosis (Fig. 5A-B). Pioglitazone has been reported to inhibit ACSL4 to protect breast cancer cells from ferroptosis [36], in parallel, pioglitazone functions as a stabilizer of CISD1 to inhibit mitochondrial iron uptake [18, 22], which dictates another mechanism of pioglitazone in the regulation of ferroptosis. We found that pioglitazone significantly reduced mitochondrial iron content as well as cellular iron levels in GCN5L1 KO cells (Fig. 5C-D). Furthermore, NL-1 accelerated sorafenib-induced ROS levels, whereas pioglitazone reduced sorafenib-induced ROS levels (Fig. 5E). Then we found that NL-1 significantly enhanced lipid peroxidation and further strengthened sorafenib-induced C11-BODIPY signals (Fig. 5F). In contrast, CISD1 agonist pioglitazone significantly inhibited cellular ROS and lipid peroxidation in GCN5L1 KO cells (Fig. 5G-H), supporting that GCN5L1 deletion increased ROS and lipid peroxidation levels via inhibition of CISD1.

### The combination of sorafenib with NL-1 accelerates sorafenib effect in vitro and in vivo

**Fig. 6:**
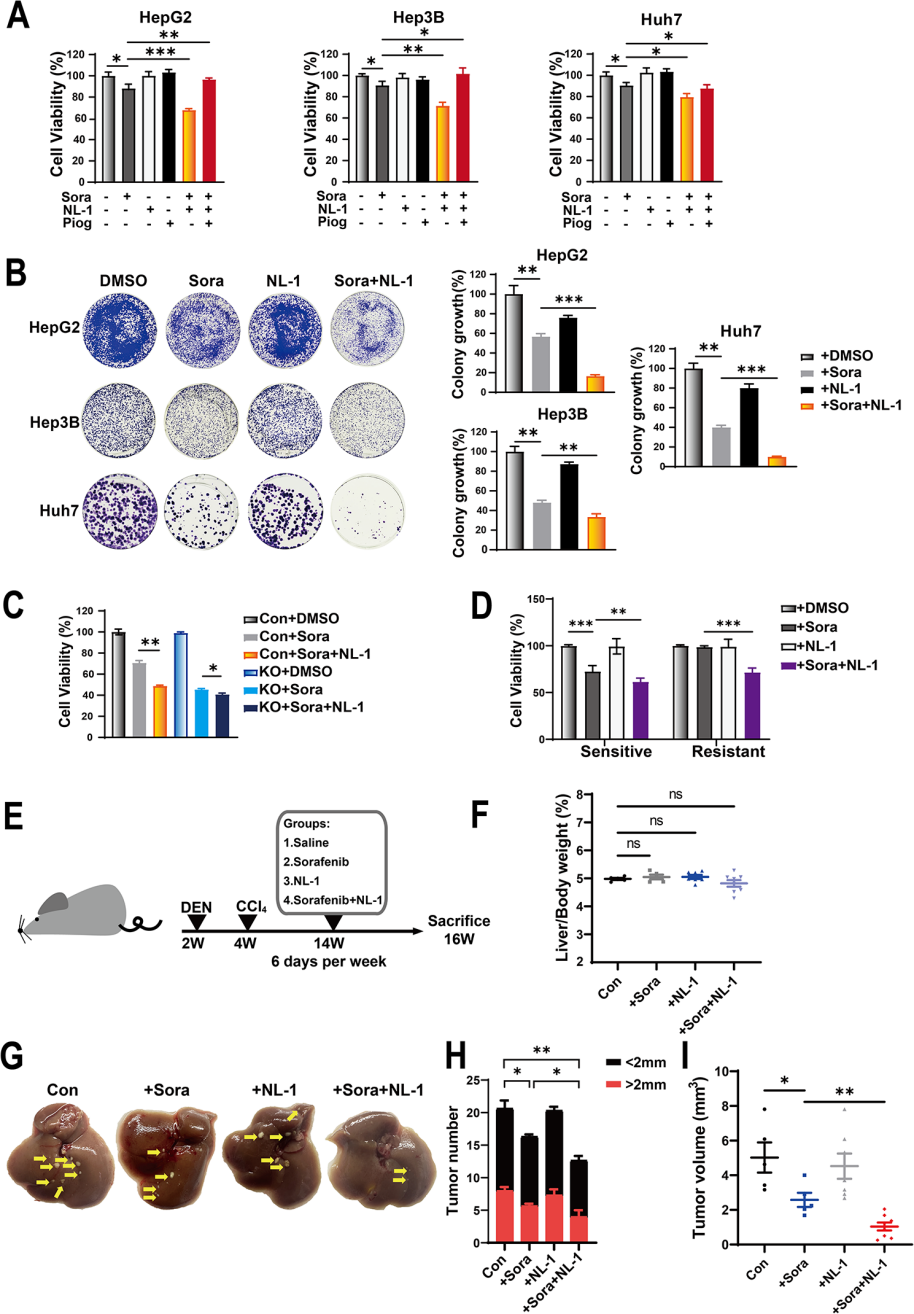
The combination of sorafenib with NL-1 accelerates sorafenib effect in vitro and vivo. (**A**) Cell viability in HCC cells (HepG2, Hep3B, Huh7) treated with or without sorafenib (5 μM), NL-1 (20 μM) and pioglitazone (10 μM) for 48 h. (**B**) The colony formation assay shown the growth effect of sorafenib (2.5 μM), NL-1 (10 μM) treated in individual or combination in HCC cells (HepG2, Hep3B, Huh7) for 14 days as well as quantification of clusters (right). (**C**) Cell viability in HepG2 cells with sorafenib (8 μM), NL-1 (20 μM) treated in individual or combination for 48 h. (**D**) Cell viability in sorafenib sensitive or resistant HepG2 cells with sorafenib (5 μM), NL-1 (20 μM) in individual or combination for 48 h. (**E**) Schematic of the experimental design. Diethylnitrosamine (DEN) plus CCl_4_-induced HCC mouse model and treatment with chemicals. (Groups: Saline *n* = 5; Sorafenib *n* = 5; NL-1 *n* = 7; Sorafenib + NL-1 *n* = 8). (**F**) Ratio of liver weight to body weight. (**G**) Representative images of mouse livers from mice with the indicated treatments. Arrows indicate tumors. (**H**) Tumor number of each group of mice. (Groups: Saline *n* = 5; Sorafenib *n* = 5; NL-1 *n* = 7; Sorafenib + NL-1 *n* = 8). (**I**) Tumor volume of each group of mice. (Groups: Saline *n* = 5; Sorafenib *n* = 5; NL-1 *n* = 7; Sorafenib + NL-1 *n* = 8). DMSO dimethyl sulfoxide, Sora sorafenib, Piog pioglitazone. Values represent the mean ± SEM of three independent experiments. The two-tailed unpaired student’s t-test or ANOVA plus a Bonferroni post hoc test were used to assess the differences between two or more groups, *p* < 0.05 indicates statistical significance. *p < 0.05, **p < 0.01 ***p < 0.001

To investigate whether the combination of NL-1 with sorafenib enhances sorafenib sensitivity, we accessed the function of NL-1 on sorafenib induced cell death. A low dose of sorafenib slightly reduced cell viability in HCC cells, the co-incubation with NL-1 significantly improved sorafenib-induced cell death (Fig. 6A-B). Again NL-1 enhanced sorafenib-induced cell death at 8 μM, which is a common dose of sorafenib, however, NL-1 barely increased sorafenib sensitivity in KO cells, suggesting GCN5L1 KO shared the same target with NL-1 (Fig. 6C). Furthermore, sorafenib resistant HepG2 cells were treated with sorafenib, NL-1 or the combination of these chemicals. Sorafenib showed little effect on resistant HepG2 inhibition, however, NL-1 significantly enhanced sorafenib sensitivity in those cells (Fig. 6D). We then generated the DEN-induced HCC mouse model and treated the mice with sorafenib, NL-1 or the combination of these chemicals (Fig. 6E). These groups of mice were observed with similar liver/body weight ratio (Fig. 6F). NL-1 treatment did not show differentiation in tumor number or tumor volume compared to vehicle treatment, however, the combination of NL-1 and Sora significantly reduced tumor number and tumor volume compared to vehicle treatment and enhanced sorafenib efficacy (Fig. 6G-I). These results implied that CISD1 was an important target of GCN5L1 to regulate the sensitivity of HCC to sorafenib, and CISD1 inhibitor in combination with sorafenib could enhance the therapeutic effect in sorafenib sensitive HCC and re-sensitize sorafenib resistant cells, which would be a potentially pharmaceutical strategy for clinical therapy.

## Discussion

Hepatocellular carcinoma (HCC) is the most common primary malignant liver tumor worldwide [37]. Surgical resection is the optimal choice for treating HCC in early stage [38]. However, due to the lack of typical symptoms in HCC early stage and the high metastatic and invasive potential of cancer cells, the optimal timing for surgical treatment is often missed for HCC patients in advanced-stage [39]. Sorafenib is the first targeted drug approved for advanced-stage HCC [14] but only about 30% of patients are sensitive to sorafenib, and most patients immediately develop drug resistance after an average of 6 months [15]. It is evident that the sensitivity of patients to drugs limits the clinical application of targeted drugs, while drug resistance leads to treatment failure [40]. Previous studies have indicated the prominent contribution of iron in ferroptosis, which requires its potential to increase the activity of enzymes responsible for lipid peroxidation and oxidative homeostasis, such as typical lipoxygenases (ALOX) [41].

Sorafenib-induced ferroptosis requires excessive ROS and iron overload, which brings mitochondria to be considered as a major mediator involved in the mechanism of sorafenib [42]. Mitochondria are the cellular hubs of ROS [43], as well as the focal point of iron metabolism and hemostasis [44]. Hepatic iron overload is related to HCC development, since iron is necessary for mitochondrial OXPHOS to provide energy for HCC survival and metastasis. On the other hand, mitochondrial iron participates in mitochondrial ROS (mito-ROS) generation [45]. Excessive mito-ROS reacts with PUFAs in the mitochondrial membrane, leading to lipid peroxidation and subsequent mtDNA damage [46]. Thus, mitochondrial iron homeostasis is critical to ferroptosis. Interestingly, sorafenib resistant cells exhibit iron deficiency, suggesting that replenishment of iron or blockage of iron depletion could be a potential strategy to re-sensitize HCC to sorafenib.

Our previous work has reported that GCN5L1 loss activates fatty acid oxidation to enhance ROS generation, independent of mitochondrial damage, which promotes HCC metastasis [25]. Interestingly, GCN5L1 loss promotes HCC proliferation [26], suggesting appropriate ROS is not sufficient to induce ferroptosis but may bring the cells to a steady state for ferroptosis. In the current study, we observed that acute sorafenib incubation significantly inhibits the expression of GCN5L1 in HCC cells, however, sorafenib resistance is associated with increased GCN5L1 expression. Our experiments revealed that GCN5L1 deleted HCC cells were more sensitive to sorafenib, while mitochondrial restricted GCN5L1 overexpression blunted sorafenib sensitivity. These data imply GCN5L1 expression is associated with sorafenib sensitivity, suggesting that GCN5L1 could be an indicator of sorafenib sensitivity for clinical application.

On the other hand, iron accumulation was observed in GCN5L1 deleted HepG2 cells via the suppression of the expression of iron release transporters, CISD1 and SLC40A1. CISD1 has been reported to be important in erastin-induced ferroptosis [22]. We observed CISD1 expression shares similar pattern as GCN5L1 in response to sorafenib acute treatment and resistance. Overexpression of mitochondrial restricted GCN5L1 restored CISD1 expression in basal condition and in response to sorafenib, suggesting mitochondrial GCN5L1 regulates CISD1 expression in a retrograde mode. CISD1 expression is associated with cellular iron contents (Supplementary Fig. 3). Increased ROS levels were observed in GCN5L1 deleted HepG2 cells due to FAO activation [25]. Mito-TEMPO or NAC, the mitochondrial ROS scavenger partially restored CISD1 expression levels in GCN5L1 deleted HepG2 cells, suggesting that CISD1 expression was susceptible to cellular redox homeostasis. Moreover, GCN5L1 deletion promotes HCC metabolic reprogramming [26] might provide metabolites for epigenetic regulation of CISD1.

Interestingly, sorafenib resistance links to iron deficiency, which seems to be associated with inhibition of iron uptake and activation of mitochondrial iron release (Figure S2), suggesting that targeting mitochondrial iron homeostasis could be crucial for sorafenib. In our current work, we found mitochondrial iron is critical for cellular iron homeostasis and sufficient to enhance sorafenib-induced ferroptosis. CISD1 inhibitor NL-1, which increases mitochondrial iron as well as cellular iron levels, sensitizes HCC cells to sorafenib. The combination of NL-1 and sorafenib significantly increases sorafenib activity for HCC treatment. Acute sorafenib treatment reduces CISD1 expression, which could be a negative effect of drugs combination to treat HCC. The combination of NL-1 and sorafenib could be more effective in sorafenib resistant HCC, since the increased expression of CISD1 in sorafenib resistant cells. Moreover, ferroptosis is closely related to the development of pulmonary and cardiovascular diseases, whether CISD1 could be a key factor in the regulation of iron homeostasis and disease related potential target is worth investigating [47].

Iron deficiency impairs fatty acid oxidation and desaturation of fatty acids but promotes lipogenesis [48], while iron overload inhibits fatty acid oxidation [49]. Thus, mitochondrial iron dynamics should be critical for the metabolic reprogramming of HCC to meet energy demand for proliferation and metastasis as well as mediating sorafenib-induced ferroptosis. HCC cells with high metastatic potential possess high rate of FAO and ROS levels [25], so it’s worth investigating the effects of sorafenib on HCC metastasis.

## Conclusions

Mitochondria are crucial in metabolic reprogram during HCC development, which suggests targeting mitochondrial metabolic pathways could be potential cancer therapy. This study demonstrates a novel regulatory pathway of mitochondrial iron homeostasis linking to sorafenib resistance. We identified GCN5L1 as a modulator in sorafenib-induced ferroptosis via targeting mitochondrial iron transporter CISD1. Low expression of GCN5L1 or CISD1 promoted ROS generation and iron accumulation, which were necessary for sorafenib induced ferroptosis. High expression of GCN5L1 and CISD1 was associated with sorafenib resistance. The combination of CISD1 inhibitor NL-1 with sorafenib enhanced sorafenib efficacy in vitro and in vivo. Therefore, GCN5L1/CISD1 axis is crucial for sorafenib resistance and would be a potential therapeutic strategy for sorafenib resistant HCC.

### Electronic supplementary material

Below is the link to the electronic supplementary material.

Supplementary Material 1

## Data Availability

All data for the duration of this study are included in this article and its accompanying documentation. The data used in this study can be obtained from corresponding authors upon reasonable request.

## Abbreviations

ALOX: Arachidonate lipoxygenase
DEN: Diethylnitrosamine
DFO: Deferoxamine
DMSO: Dimethyl sulfoxide
FAC: Ammonium iron citrate
Fer-1: Ferrostatin-1
MFRN1/2: Mitoferrin1/2
MtG: Mitochondrial localized GCN5L1
NAC: N-acetylcysteine
Nec-1: Necrostatin-1
Piog: Pioglitazone
PUFAs: Polyunsaturated fatty acids
ROS: Reactive oxygen species
Sora: Sorafenib
TF: Transferrin
TFR1: Transferrin receptor 1
Z-VAD: Z-VAD-FMK
