# Supplementary material for: Mitochondrial GCN5L1 acts as a novel regulator for iron homeostasis to promote sorafenib sensitivity in hepatocellular carcinoma
Source: J Transl Med. 2024 Jun 25;22:593. doi: 10.1186/s12967-024-05404-3 (PMC11201091; doi:10.1186/s12967-024-05404-3)
Supplement: Supplementary file 1 — Supplementary Material 1 [file 12967_2024_5404_MOESM1_ESM.docx]

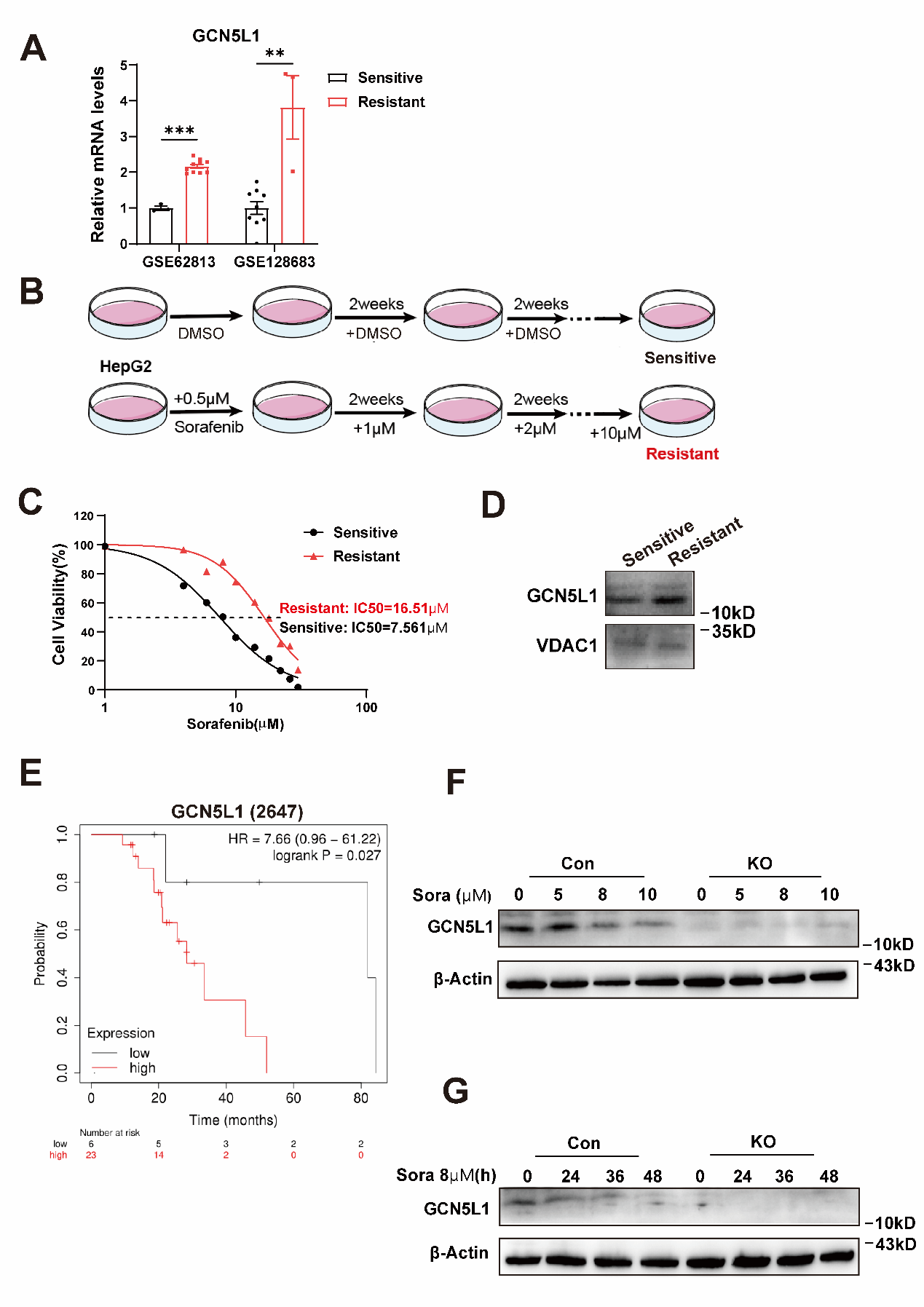


**Supplementary Figure 1. GCN5L1 is associated with HCC drug resistance.**

1. GCN5L1 mRNA level in GEO dataset GSE62813 and GSE128683. GSE62813: Sorafenib-sensitive HepG2 cells (n = 3) and -resistant cells (n = 10). GSE128683: Sorafenib-sensitive HepG2 cells (n = 9) and -resistant cells (n = 3).
2. The establishment of sorafenib sensitive/resistant HepG2 cells
3. The IC50 values of sorafenib-sensitive cells treated with sorafenib (up). CISD1 protein level in sorafenib sensitive and resistant cells (below).
4. GCN5L1 protein levels in sorafenib-sensitive and -resistant cells.
5. Overall survival of LIHC patients with sorafenib treatment, stratified by GCN5L1 expression.
6. Expression of GCN5L1 protein in cells treated with 0, 5, 8, 10 μM of sorafenib as detected by immunoblotting.
7. Expression of GCN5L1 protein in cells treated with 8 μM of sorafenib for 0, 24, 36 or 48 h, as measured by immunoblotting.

Values represent the mean ± SEM of three or more independent experiments. Statistical significance was calculated using two-tailed unpaired Student’s t-test. *p < 0.05, **p < 0.01 ***p < 0.001.


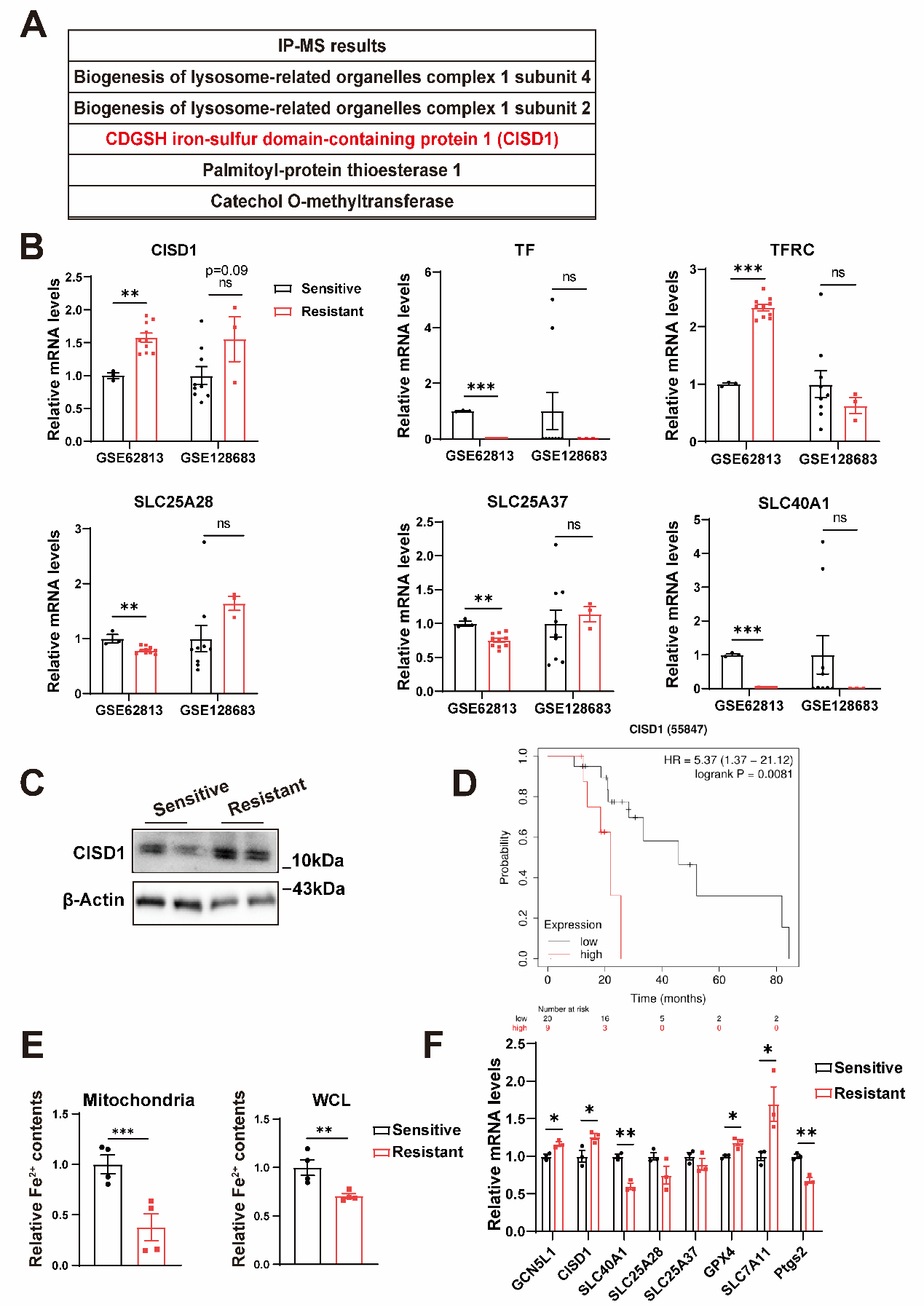


**Supplementary Figure 2. CISD1 is identified as a target gene regulated by GCN5L1 in ferroptosis.**

IP-MS results were analyzed for the top mitochondrial proteins of GCN5L1 interaction.

Iron transporter genes mRNA levels in GEO dataset GSE62813 and GSE128683. GSE62813: Sorafenib-sensitive HepG2 cells (n = 3) and -resistant cells (n = 10). GSE128683: Sorafenib-sensitive HepG2 cells (n = 9) and -resistant cells (n = 3).

CISD1 protein expression level in sensitive and resistant HepG2 cells.

Overall survival of LIHC patients with sorafenib treatment, stratified by CISD1 expression.

The mitochondrial and total iron levels in sorafenib sensitive and resistant HepG2 cells (n=4).

RT-qPCR analyses of ferroptosis relative gene expression in sorafenib sensitive and resistant HepG2 cells (n=3).

Values represent the mean ± SEM of three or more independent experiments. Statistical significance was calculated using two-tailed unpaired Student’s t-test. *p < 0.05, **p < 0.01 ***p < 0.001.


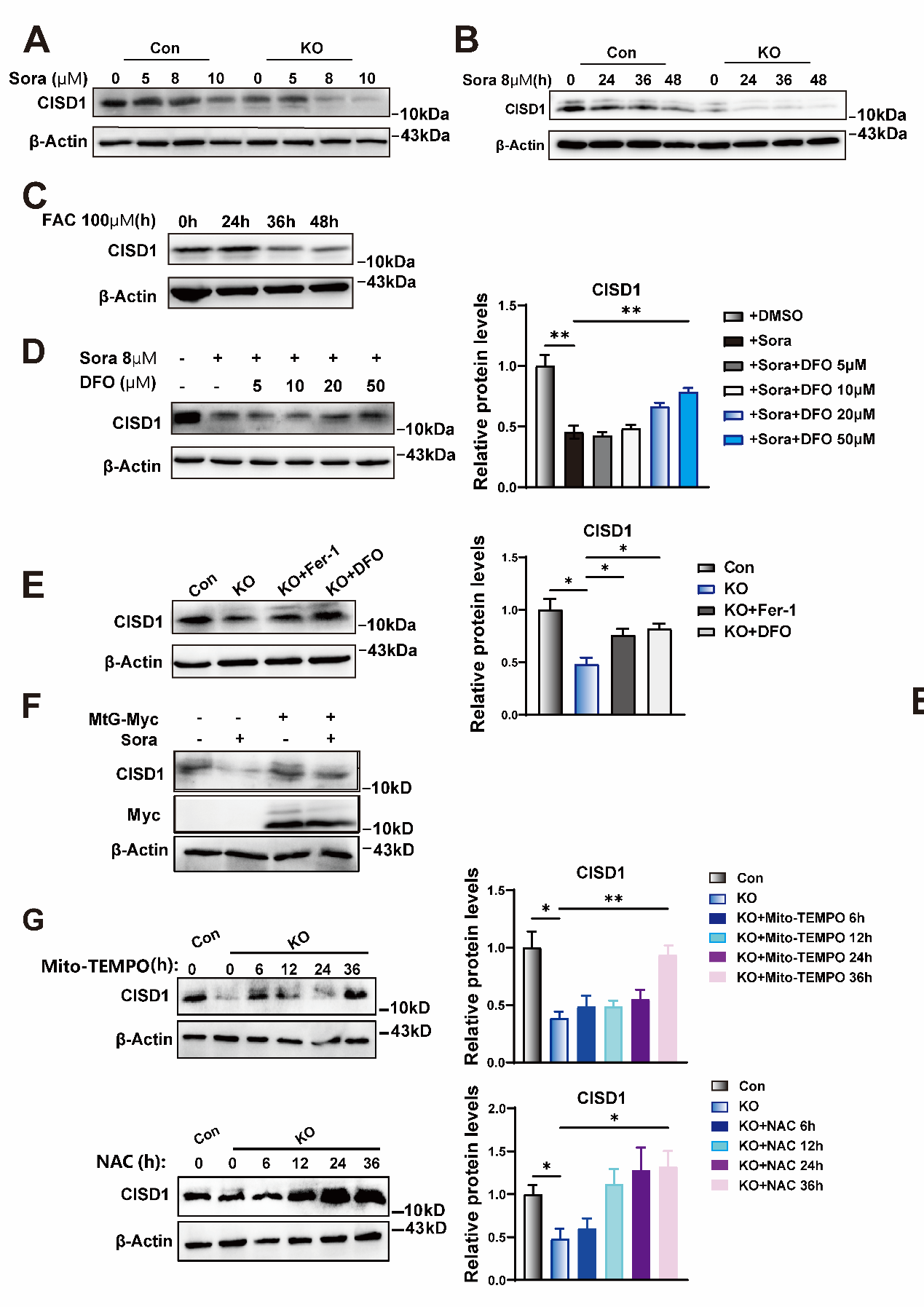


**Supplementary Figure 3. CISD1 was susceptible to acute sorafenib treatment.**

1. Expression of CISD1 protein in HepG2 cells treated with 0, 5, 8, 10 μM of sorafenib as detected by immunoblotting.
2. Expression of CISD1 protein in HepG2 cells treated with 8 μM of Sora for 0, 24, 36 or 48 h, as measured by immunoblotting.
3. Expression of CISD1 protein in HepG2 cells treated with or without FAC in indicated concentrations for 24 h.
4. Expression and quantification of CISD1 protein levels in HepG2 cells treated with 8 μM of sorafenib with or without DFO in indicated concentrations for 24 h.
5. Expression and quantification of CISD1 protein levels after HepG2 cells were pretreated with Fer-1 (10 μM), DFO (100 μM) for 24h.
6. Expression of CISD1 protein in HepG2 cells after overexpression of mitochondrial restricted GCN5L1 (MtG-Myc) and then treated with/without sorafenib (10μM) for 24h.
7. Expression and quantification of CISD1 protein levels after HepG2 Con/KO cells were treated with/without Mito-TEMPO (20 μM) or NAC (200 μM) for 6, 12, 24, 36h.

Sora, sorafenib; FAC, ammonium iron citrate (Fe^3+^); DFO, deferoxamine; Fer-1, ferrostatin-1; NAC, N-acetylcysteine. Values represent the mean ± SEM of three or more independent experiments. Statistical significance was calculated using two-tailed unpaired Student’s t-test. *p < 0.05, **p < 0.01 ***p < 0.001.

Supplementary Table 1. The sequences of RT-PCR primers.

| Gene | Forward primer | Reverse primer |
| --- | --- | --- |
| β-Actin | CCAGCCTTCCTTCTTGGGTA | CAATGCCTGGGTACATGGTG |
| GCN5L1 | AAGAACACCAAGCCAAGCAG | GTTGAGGTGATCCACCAACG |
| CISD1 | GCCTTCGACATGGAGGATCT | ATCGCAGAAGGGGAACTTTT |
| SLC40A1 | CCGCTTCCATAAGGCTTTGC | TGACTGGGGAGCCAAATGTC |
| TF | AGAAGCGAGTCCGACTGTG | TATGGTCGCGGAAACTCTGG |
| TFRC | CATATGTCCCTCGTGAGGCT | GCGCTGTCTTTGACCTGAAT |
| MFRN | GCACGCCATGTATTTTGCCT | CTCCCAGCTACCCCATTAGC |
| MFRN2 | CCACTGTCACCACGCACAT | CTGCATCCGGGTCTTGACG |
| Me1 | AGTTTGGTGTTTCGGAAGCC | TACAGCCAAGGTCTCCCAAG |
| SLC7A11 | CCTCTGCCAGCTGTTATTGTT | CCTGGCAAAACTGAGGAAAT |
| GPX4 | GCCTGGATAAGTACAGGGGTT | CATGCAGATCGACTAGCTGAG |

Supplementary Table 2. The results of IP-MS.

| **Accession** | **Description** | **ΣCoverage** |
| --- | --- | --- |
| P78537 | Biogenesis of lysosome-related organelles complex 1 subunit 1 OS=Homo sapiens GN=BLOC1S1 PE=1 SV=2 - [BL1S1_HUMAN] | 55.56 |
| Q5XKP0 | MICOS complex subunit MIC13 OS=Homo sapiens GN=MIC13 PE=1 SV=1 - [MIC13_HUMAN] | 54.24 |
| P98179 | RNA-binding protein 3 OS=Homo sapiens GN=RBM3 PE=1 SV=1 - [RBM3_HUMAN] | 31.85 |
| O95295 | SNARE-associated protein Snapin OS=Homo sapiens GN=SNAPIN PE=1 SV=1 - [SNAPN_HUMAN] | 29.41 |
| Q96GS4 | BLOC-1-related complex subunit 6 OS=Homo sapiens GN=BORCS6 PE=1 SV=2 - [BORC6_HUMAN] | 28.29 |
| P51149 | Ras-related protein Rab-7a OS=Homo sapiens GN=RAB7A PE=1 SV=1 - [RAB7A_HUMAN] | 25.12 |
| P10599 | Thioredoxin OS=Homo sapiens GN=TXN PE=1 SV=3 - [THIO_HUMAN] | 20.95 |
| O00264 | Membrane-associated progesterone receptor component 1 OS=Homo sapiens GN=PGRMC1 PE=1 SV=3 - [PGRC1_HUMAN] | 20.51 |
| Q9NUP1 | Biogenesis of lysosome-related organelles complex 1 subunit 4 OS=Homo sapiens GN=BLOC1S4 PE=1 SV=1 - [BL1S4_HUMAN] | 20.28 |
| Q6QNY1 | Biogenesis of lysosome-related organelles complex 1 subunit 2 OS=Homo sapiens GN=BLOC1S2 PE=1 SV=1 - [BL1S2_HUMAN] | 19.01 |
| Q99439 | Calponin-2 OS=Homo sapiens GN=CNN2 PE=1 SV=4 - [CNN2_HUMAN] | 18.77 |
| Q9UL25 | Ras-related protein Rab-21 OS=Homo sapiens GN=RAB21 PE=1 SV=3 - [RAB21_HUMAN] | 17.33 |
| O43809 | Cleavage and polyadenylation specificity factor subunit 5 OS=Homo sapiens GN=NUDT21 PE=1 SV=1 - [CPSF5_HUMAN] | 16.74 |
| P82650 | 28S ribosomal protein S22, mitochondrial OS=Homo sapiens GN=MRPS22 PE=1 SV=1 - [RT22_HUMAN] | 16.67 |
| Q92600 | CCR4-NOT transcription complex subunit 9 OS=Homo sapiens GN=CNOT9 PE=1 SV=1 - [CNOT9_HUMAN] | 16.39 |
| P63172 | Dynein light chain Tctex-type 1 OS=Homo sapiens GN=DYNLT1 PE=1 SV=1 - [DYLT1_HUMAN] | 15.93 |
| Q9HD34 | LYR motif-containing protein 4 OS=Homo sapiens GN=LYRM4 PE=1 SV=1 - [LYRM4_HUMAN] | 15.38 |
| O43504 | Ragulator complex protein LAMTOR5 OS=Homo sapiens GN=LAMTOR5 PE=1 SV=1 - [LTOR5_HUMAN] | 15.38 |
| P51808 | Dynein light chain Tctex-type 3 OS=Homo sapiens GN=DYNLT3 PE=1 SV=1 - [DYLT3_HUMAN] | 14.66 |
| P18859 | ATP synthase-coupling factor 6, mitochondrial OS=Homo sapiens GN=ATP5J PE=1 SV=1 - [ATP5J_HUMAN] | 13.89 |
| Q9NZ45 | CDGSH iron-sulfur domain-containing protein 1 OS=Homo sapiens GN=CISD1 PE=1 SV=1 - [CISD1_HUMAN] | 13.89 |
| P50897 | Palmitoyl-protein thioesterase 1 OS=Homo sapiens GN=PPT1 PE=1 SV=1 - [PPT1_HUMAN] | 13.73 |
| P21964 | Catechol O-methyltransferase OS=Homo sapiens GN=COMT PE=1 SV=2 - [COMT_HUMAN] | 13.65 |
| Q9HB71 | Calcyclin-binding protein OS=Homo sapiens GN=CACYBP PE=1 SV=2 - [CYBP_HUMAN] | 13.60 |
| O14561 | Acyl carrier protein, mitochondrial OS=Homo sapiens GN=NDUFAB1 PE=1 SV=3 - [ACPM_HUMAN] | 13.46 |
| Q9P2B4 | CTTNBP2 N-terminal-like protein OS=Homo sapiens GN=CTTNBP2NL PE=1 SV=2 - [CT2NL_HUMAN] | 13.46 |
| P62995 | Transformer-2 protein homolog beta OS=Homo sapiens GN=TRA2B PE=1 SV=1 - [TRA2B_HUMAN] | 12.85 |
| Q9NY12 | H/ACA ribonucleoprotein complex subunit 1 OS=Homo sapiens GN=GAR1 PE=1 SV=1 - [GAR1_HUMAN] | 12.44 |
| Q9BT17 | Mitochondrial ribosome-associated GTPase 1 OS=Homo sapiens GN=MTG1 PE=1 SV=2 - [MTG1_HUMAN] | 12.28 |
